# Incidental Malignant Colonic Polyp Detected in a Resected Ischaemic Large Bowel: A Case Report and Literature Review

**DOI:** 10.7759/cureus.13928

**Published:** 2021-03-16

**Authors:** Philip Idaewor, Omotara Lesi, Mariam Elremeli, Noreen Rasheed, Abdalla Saad Abdalla Al-Zawi

**Affiliations:** 1 Cellular Pathology, Basildon and Thurrock University Hospital, Basildon, GBR; 2 General and Colorectal Surgery, Basildon University Hospital, Essex, GBR; 3 Allergy/Immunology, Sidra Medicine & Research Institute, Doha, QAT; 4 Pediatrics, Imperial College, London, GBR; 5 Breast Radiology, Basildon and Thurrock University Hospital, Basildon, GBR; 6 General & Breast Surgery, Basildon and Thurrock University Hospital, Basildon, GBR; 7 General & Breast Surgery, Anglia Ruskin University, Chelmsford, GBR; 8 General & Breast Surgery, Mid and North Essex University Hospital Group, Basildon, GBR

**Keywords:** malignant colonic polyp, colonic cancer, colorectal cancer, adenocarcinoma, laparotomy, lynch syndrome

## Abstract

Most patients with bowel cancer are symptomatic at the time of the diagnosis. They may present with a change in bowel habit, bleeding per rectum, abdominal pain, anaemia, weight loss or bowel obstruction. Colonic carcinoma can also be diagnosed incidentally during screening programs. Moreover, it may be incidentally detected in CT scans being performed for other indications or encountered during surgery for other causes. Some patients with colonic bowel ischaemia have associated large bowel cancer, where the ischaemic segment is usually proximal to the tumour and not necessarily associated with bowel obstruction. We are presenting a rare case of incidental malignant colonic polyp detected in a resected ischaemic large bowel in an 88-year-old gentleman. This was a very small tumour that was not visible macroscopically or detectable by imaging. Pathological examination of non-tumour colorectal resection specimens, as in this case, should include careful macroscopic examination and sequential block selection along the length of the colon, and where there is diffuse mucosal abnormality, block selection at 100mm interval is also advised. Attention to and block selection from any suspicious-looking area is warranted in all cases of non-tumour colorectal resections if such microscopic-sized malignancies of the type seen in our patient are to be picked up.

## Introduction

Colonic malignancy occupies fourth place among the most common cancers in the UK and is regarded as the second most frequent cause of cancer-related mortality. Every year, more than 42,000 new cases are diagnosed, and currently around 268,000 people are living with colonic cancer in the UK. The diagnosis of colonic cancer is usually made by histological examination of a biopsy that is gotten during colonoscopy or from a surgical specimen, with the vast majority of tumours of the colon being carcinomas. Of the carcinomas, more than 90% are adenocarcinomas [1]. Incidental colorectal carcinoma is defined as a clinically inapparent carcinoma of the large intestine diagnosed or discovered unexpectedly. It can be detected in imaging for other reasons [2], during colonoscopy bowel cancer screening [3], during surgical intervention for unrelated causes, as well as in autopsy. In this case, it was discovered during pathologic examination of a subtotal colectomy specimen removed for ischaemic colitis. The prevalence of incidentally discovered colon carcinoma, as well as the molecular patterns of these incidental tumours in the UK, is underreported, and as such its importance in the evaluation of the incidence and future trends of colon cancers is currently unknown.

## Case presentation

**Figure 1:**
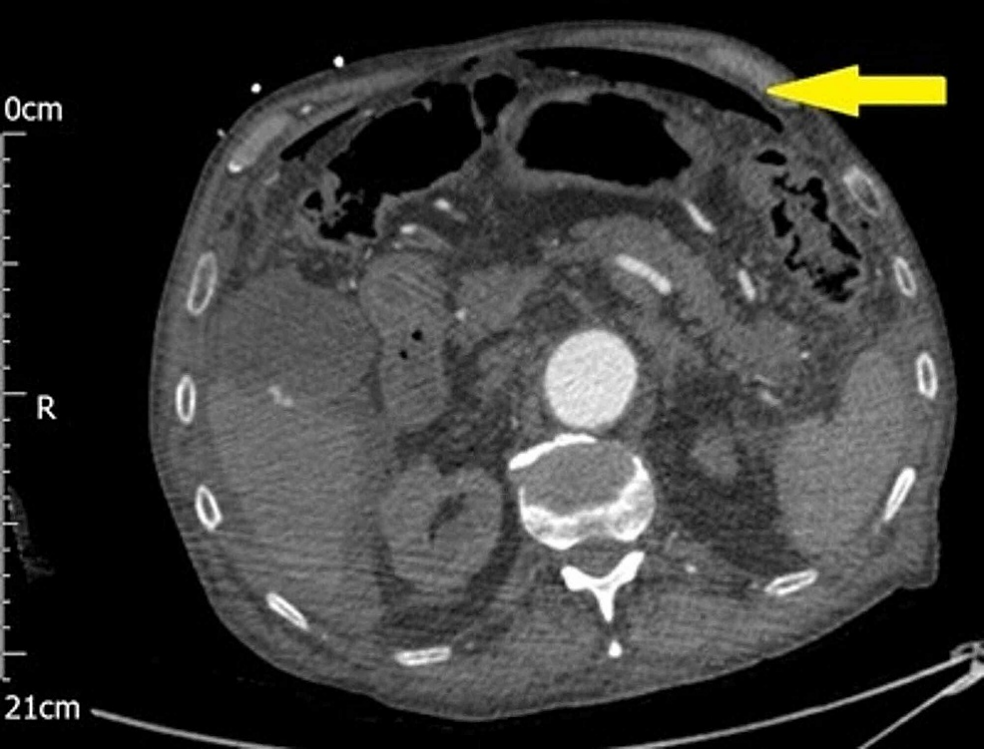
CT abdomen axial view shows free intra-peritoneal air (yellow arrow)

**Figure 2:**
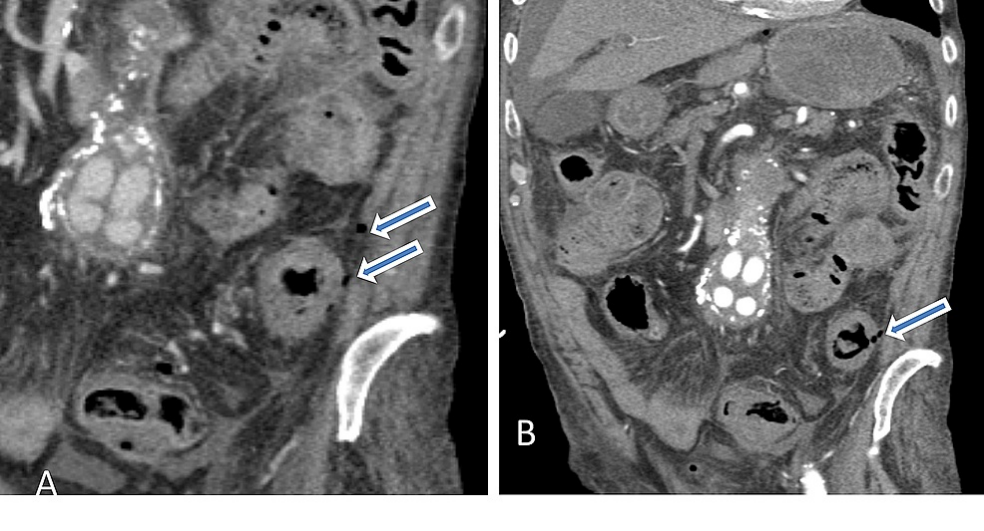
Abdominal contrast-enhanced coronal images demonstrates circumferential thickening of the descending colon with peri colonic fat stranding (A) and evidence of a few extra luminal gas locules suggesting possible site of perforation as indicated by blue arrows (A&B). There are multiple contrast-enhancing rounded structures in the root of mesentery, they represent aortobiiliac stent within the abdominal aortic aneurysm.

**Figure 3:**
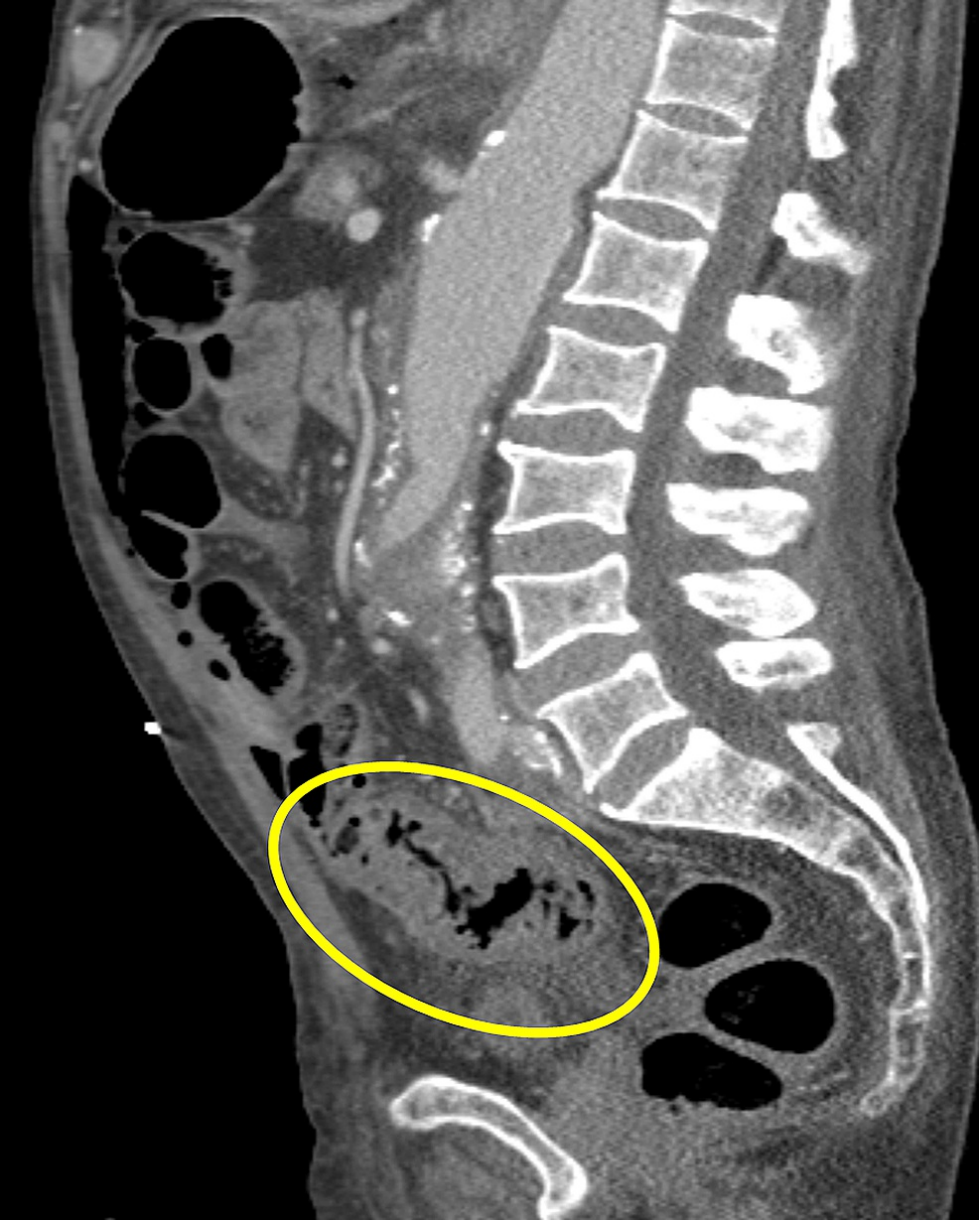
Contrast-enhanced sagittal image demonstrates diffuse circumferential thickening of the descending and proximal sigmoid colon with associated pericolonic fat stranding, in keeping with ongoing colitis. Considering the limitation due to unprepared CT scan for bowel loops evaluation, possible small intraluminal sinister polyp or mass cannot be excluded.

**Figure 4:**
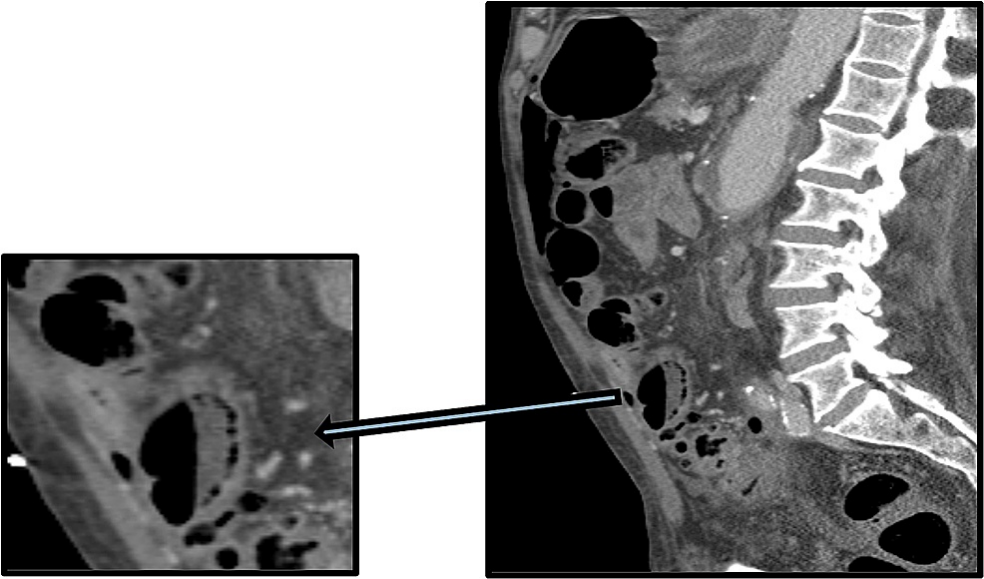
Contrast-enhanced sagittal image with magnified view demonstrates gas locules within the bowel loop wall in keeping with pneumatosis intestinalis, which is suggestive of ischaemic bowel.

An 88-year-old male presented with a recent history of acute abdominal pain and vomiting. His past medical history included ischaemic heart disease, cerebrovascular accident and an open repair of abdominal aortic aneurysm 10 years earlier, in addition to an unoperated incisional hernia. On examination, the patient was septic and in cardiogenic shock. Urgent CT abdomen and pelvis showed free intra-abdominal air (Figure 1), thickened descending and proximal sigmoid colon with locules of transmural air, as well as locules of extra-luminal gas around it, and this was thought to be the site of the perforation. Because of the presence of aortobiiliac stent within the abdominal aortic aneurysm, origin of the inferior mesenteric artery could not be delineated. However normally opacified tributaries of inferior mesenteric artery supplying the sigmoid and descending colon via collaterals were demonstrated (Figures 2, 3, 4). The patient had undergone an emergency laparotomy, adhesiolysis and subtotal colectomy with an end ileostomy. Intra-operative findings included friable large bowel from mid-sigmoid to caecum with a widespread ischaemic appearance and multiple perforations. Postoperatively, the patient required intensive care unit admission, and unfortunately, he died a week later due to sepsis and multiple organ failure.

**Figure 5:**
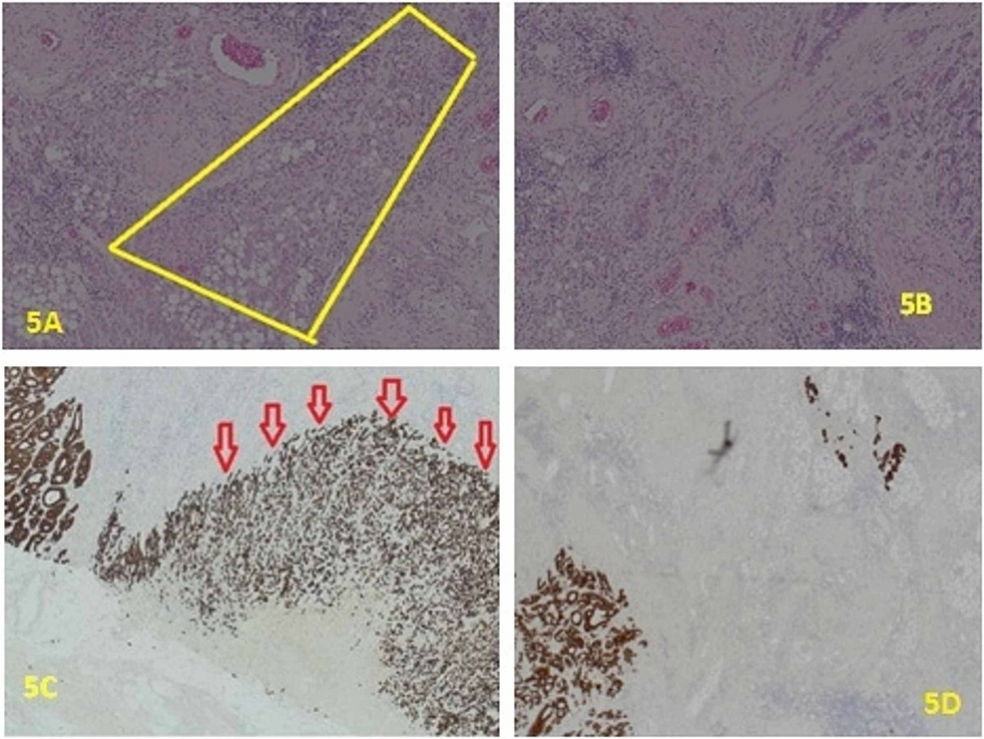
A: H&E x10. pT3 (poorly differentiated sheet of tumour cells infiltrating pericolic adipose tissue). B: H&E x10. Cords and sheet of moderately differentiated and poorly differentiated tumour are seen in section. C: x2. Pancytokeratin (MNF116) highlighting poorly differentiated tumour component both superficially and deeply in the fat of the colon wall (red arrows). D: x4. Pancytokeratin (MNF116) highlighting tumour cells both superficially and deeply in the fat of the colon wall. H&E: hematoxylin and eosin

**Figure 6:**
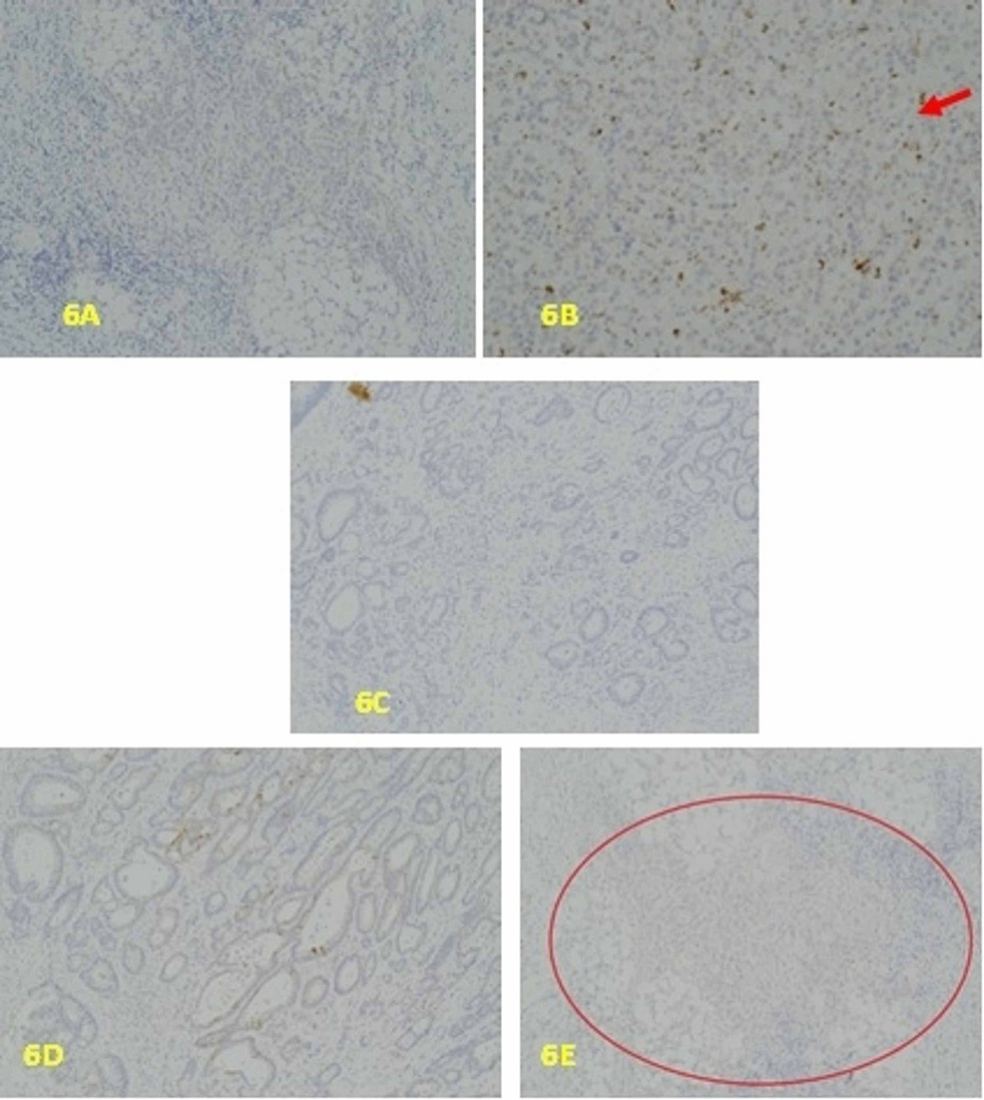
A: x10, CDX2 uniformly negative in the poorly differentiated tumour comonent. B: x10. The poorly differentiated tumour cells are negative with CD68 immunostaining which highlights the tumour associated macrophages, as indicated by the red arrow. C: x10. The tumour is completely negative for CK7. D: x10. CK20 Showing patchy positivity of tumour in the better differentiated superficial aspect of the tumour. E: x10. CK20 The poorly differentiated aspect of the tumour is uniformly negative. The sheets of tumour cells are mostly within the red circled area.

Pathological examination of the resected bowel revealed colectomy with terminal ileum specimen; the appendix was not identified. On opening the colon, there were extensive but patchy areas of mucosal ulceration with elevated edges. The margins of the ulcers appeared elevated. A focus of colonic perforation was identified. No tumours were identified macroscopically. The mucosa was extensively sampled sequentially from the ileum through the distal colon to include both resection margins. Microscopic examination of the ileum appeared normal, while sections from the large bowel showed extensive areas of mucosal ulceration with features of transmural acute ischaemic necrosis and associated suppurative serositis. Arteriosclerotic vascular changes in several blood vessels with some vessels showing marked luminal narrowing supportive of ischaemic colitis are noted. A single 8mm polyp of mainly poorly differentiated adenocarcinoma in addition to well-differentiated adenocarcinoma that had invaded into the pericolic adipose tissue (pT3) was found in a single section from the ascending colon. The poorly differentiated adenocarcinoma was present in both the mucosal surface and deeper layers of the colonic wall beyond the muscularis propria (Figure 5A, 5B). The full extent of tumour invasion by the poorly differentiated component was highlighted by pancytokeratin (MNF116) immunostaining (Figure 5C, 5D). CDX2 was positive in the well-differentiated component of the tumour but uniformly negative in the poorly differentiated aspect (Figure 6A). In the pericolic fat, the tumour cells were differentiated from macrophages with CD68 that stained the macrophages (Figure 6B). The poorly differentiated component of the tumour in our case showed negativity for CK7 uniformly. CK20 showed patchy positivity in the superficial well-differentiated component but was uniformly negative in the poorly differentiated component. The primary colonic origin of the adenocarcinoma, especially the poorly differentiated component, necessitated immunostaining with CK7 (Figure 6C), which was negative. CK20, which is an epithelial marker with restricted expression compared to CK7, revealed very occasional positive tumour cells in the well-differentiated component (Figure 6D), but was uniformly negative in the poorly differentiated component (Figure 6E). Special AT-rich sequence-binding protein 2 (SATB2), a useful new highly specific biomarker, is expressed in 85% of all colorectal carcinomas. In combination with CK20, it identifies more than 95% of all colorectal cancers; it is recommended in lower GI tumours due to its high specificity. The above immunohistochemical pattern is consistent with the established practice from molecular studies that colorectal cancer is a heterogeneous group of neoplastic diseases that develop through three main pathogenetic pathways, namely the chromosomal instability pathway, the microsatellite instability (MSI) pathway, and the CpG island methylation pathway. Clinicopathologic studies are also pointing to colorectal carcinomas originating through these three pathways to differ in the type of precursor lesions, natural history, and pathological features. The combination of CK20/CK7 immunoprofiles showed that the CK20+/CK7− profile was the highest (60.4%), CK20−/CK7− was 35.4%, CK20+/CK7+ was 2.1% and CK20−/CK7+ was 2.1%. CK20 is specific for colonic, urothelial and Merkel cell carcinoma. On the other hand, CK7 is characteristic of glandular malignancies originating from the breast, respiratory tract, biliary tract and Mullerian epithelium [4].

**Figure 7:**
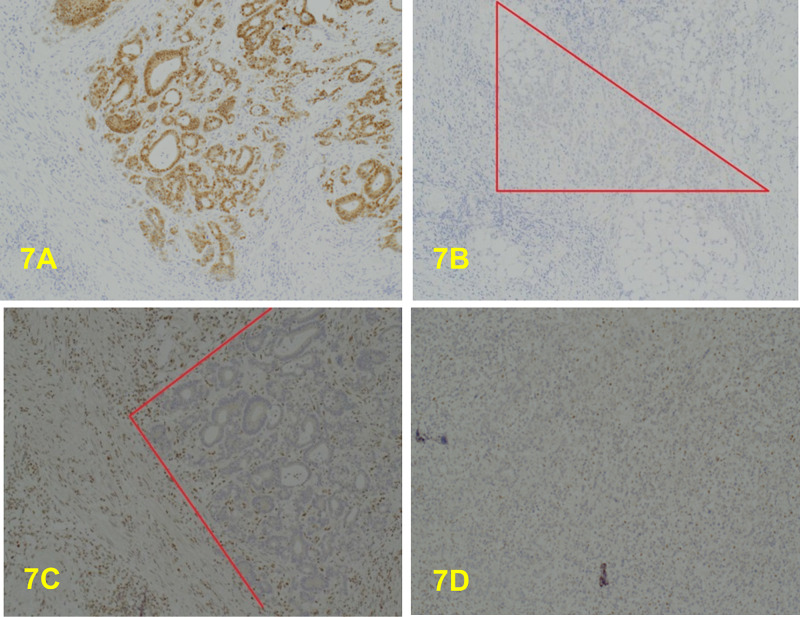
A: x10. CDX2 is uniformly positive in the superficial well/moderately differentiated aspect of the tumour. B: x10. CDX2. The poorly differentiated component of the tumour is uniformly negative, mostly within the marked area. C: x4. MLH1-negative tumour (well/moderately differentiated area of glandular elements within the red marked area). D: x4. MLH1 is also diffusely negative in the poorly differentiated component.

So CK7−/CK20− pattern as seen in the tumour we report is a known phenotype for colorectal cancer, which is now being shown to be heterogeneous in its molecular profile and therefore patient outcome [5]. CDX2 immunostaining was diffusely positively in the well-differentiated tumour component, while it was negative in the poorly differentiated component (Figure 7A, 7B). CDX2 is a homeobox gene comparatively specific for intestinal epithelial tissue [6].

**Figure 8:**
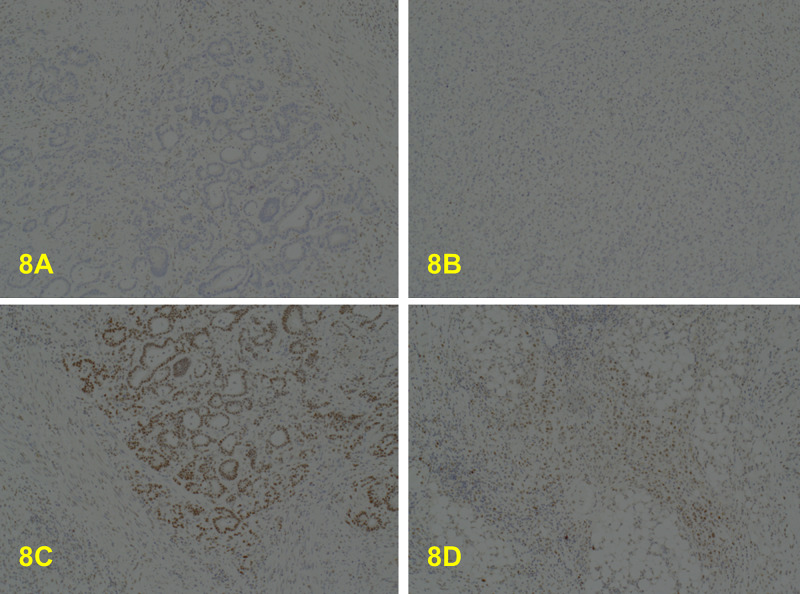
A: x4. PMS2 negative in the well/moderately differentiated component. B: x10. PMS2 also negative in the poorly differentiated component. C: x10. MSH2 positive in the well/moderately differentiated component. D: x10. MSH2 is also positive in the poorly differentiated component.

**Figure 9:**
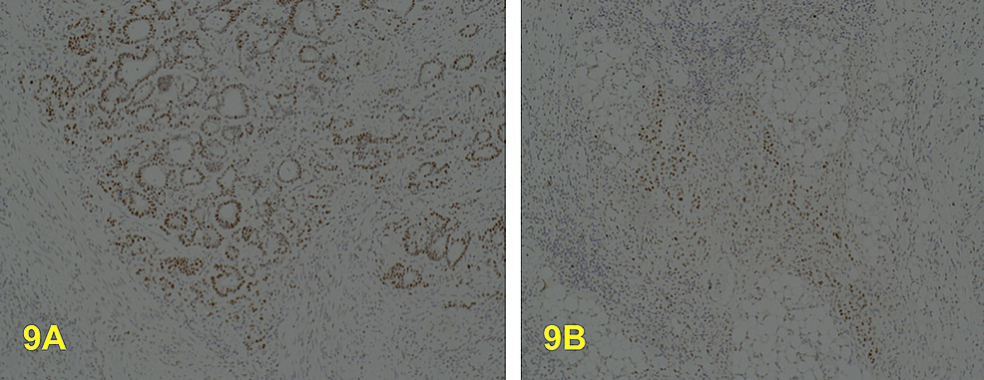
A: x10. MSH6 is positive in the well/moderately differentiated component. B: x10. MSH6 is also positive in the poorly differentiated component.

This tumour was shown by immunohistochemistry to be MMR protein deficient (MHL1 & PSM2 negative) (Figure 7C, 7D, 8A, 8B), but MSH2 (Figure 8C, 8D) and MSH6 (Figure 9A, 9B) positive. The MLH1 promoter hypermethylation was detected in the tumour-derived DNA component of the sample and within the spectrum of MSI-H and/or mismatch repair deficient (dMMR by IHC) primary colorectal adenocarcinoma, and all these results indicate a somatic process. However, these results do not exclude Lynch syndrome in a patient with a strong family history (Amsterdam II Criteria), in which germline DNA testing may still be considered necessary. This case is regarded as a de-novo condition as there is no relevant previous family history; this is significant for the other related family member.

## Discussion

The risk factors for colorectal cancer can be divided into three groups: sporadic, inherited and familial. Sporadic cases account for 70% of colorectal cancer patients and are due to dietary and environmental factors. Familial cases with known genetic syndromes make up about 5% of patients and can be divided into those with/without polyposis, while total inherited cases account for 20-30% of all colorectal cancer patients [7]. Colorectal cancer commonly presents with changes in bowel habit, abdominal pain/mass, unexplained rectal bleeding, abdominal distension, iron deficiency anaemia, unintentional weight loss or bowel obstruction. In Western countries, colorectal cancer is the third most diagnosed malignancy in males and the second in females and accounts for about 700,000 deaths per year. Most colorectal cancers display chromosomal instability and the pathogenesis follows the classical adenoma-carcinoma progressive pathway [8]. However, some colorectal carcinomas originate in non-polypoid colorectal tumours (NPT) with a lateral spreading growth pattern. These are usually a challenge and difficult to identify at colonoscopy [9]. More than 90% of colorectal cancer patients are symptomatic at the time of the diagnosis, however this disease could also be detected incidentally. Colonic carcinoma may be encountered during screening colonoscopy, which is offered to people age 55 years and over in the UK. The Danish screening program paper from 2015 revealed that the rate of incidentally detected malignant polyps was 2.3% [3]. It is also reported as an incidental finding in CT or PET scans being performed for other indications [2]. Incidental large bowel malignancy also has been noticed during surgery for other causes as in resected inguinal hernia sac, or during laparotomy for intestinal foreign body [10], and it was also detected in resected bowel after gunshot injuries [11]. Lee et al. reported that the prevalence of incidental colonic malignancy in autopsy is about 3% [12]. It has been reported that 10% of patients with colonic ischaemia have an associated large bowel malignancy, where the ischaemic segment is usually proximal to the tumour and not necessarily associated with bowel obstruction [13]. The previous reports mentioned that in the larger cancers, the blood flow in the colonic vessels could be compromised as a consequence of increased intraluminal pressure due to obstruction, which may result in bowel ischaemia, and this phenomenon is seen in 5% of patients with ischaemic colitis [14]. In our case, other factors such as sepsis, ischaemic heart disease, vascular disease and previous abdominal aortic aneurysm may have contributed to the development of bowel ischaemia.

There are three main molecular pathways in the development of colorectal cancers. First is the chromosomal instability pathway which is involved in up to 80% of colorectal carcinomas. It results from gain of function mutations which can lead to activation of oncogenes, or decreased activity of tumour suppressor genes. Mutations in APC and genes that activate the Wnt pathway are included in this pathway. The tumours are characterized by gross chromosomal abnormalities including deletions, insertions, and loss of heterozygosity. The second molecular pathogenetic pathway is the microsatellite instability (MSI) pathway where the presence of microsatellite instability accounts for about 12% of sporadic cases of colorectal cancers. This is seen with the detection of two or more unstable markers during testing for mismatch repair deficiency using a polymerase chain reaction (PCR)-based assay, or the detection of the affected protein gene products by immunohistochemistry for MMR protein products (MLH1, PMS2, MSH2 & MSH6). The third pathway is the CpG island hypermethylation pathway and was first elucidated in 1999 by Suzuki [15]. It involves gene promoter-associated CpG islands which are required for controlling transcription. When hypermethylation of these genes occurs, there is silencing of tumour suppressor genes with occurrence of tumours showing CpG island hypermethylation phenotype (CIMP). It is an epigenetic-related activity leading to instability of the CpG islands, hypermethylation of promoter CpG island sites, and the resultant inactivation of several tumour suppressor genes or other tumour-related genes. Most colorectal carcinomas with this phenotype present as flat, non-adenomatous mucosal lesions, usually in the right colon, and the sessile serrated adenomas or polyps represents an example of these lesions which have been associated with increased risk of developing colorectal carcinomas [16].

Premalignant lesions for colon cancers are local tumours that precede the development of cancers. Overwhelming evidence points to adenomas and this is referred to as the adenoma-carcinoma sequence. The adenomas could be polypoid (sessile or pedunculated), flat or depressed lesions of epithelial origin and the concerning histology feature is dysplasia. Other potential premalignant tumours include hyperplastic polyps, hamartomatous polyps, aberrant crypt foci and dysplasia in inflammatory bowel disease [17]. The particular tumour detected in this case report appears to arise from flat or sessile adenoma. The tumour detected in the colonic specimen was poorly differentiated with deficient MMR (with loss of MLH1 and PMS2) as detected through immunohistochemistry. In patients with loss of MLH1 staining on IHC, testing for acquired BRAF mutations and MLH1 promoter hypermethylation is normally done to distinguish between colorectal cancers that have loss of MLH1 that arise from Lynch syndrome (no MLH1 hypermethylation) and sporadic colorectal cancer caused by epigenetic methylation of MLH1 [18]. Also, in this tumour, MHL1 promoter hypermethylation was detected, which is consistent with localized epigenetic inactivation of the MHL1 gene, i.e. sporadic MSI-H colorectal cancer. These sporadic MSI-H colon cancers often arise in the setting of a specific pathway of DNA hypermethylation, known as the CpG island methylator phenotype (CIMP), with CIMP-related silencing of MLH1 [19]. Patients with MSI-H tumours have distinct clinical and pathological features, irrespective of their germline or sporadic origins and these include proximal colon predominance, frequent poor differentiation and mucinous histology, and increased number of tumour-infiltrating lymphocytes. In addition to being molecularly distinct from Lynch syndrome cases, patients with sporadic MSI-H cancers have associated epidemiological features, including older age at diagnosis, female gender and cigarette smoking [19]. Checking mismatch repair status in patients diagnosed with colonic cancer helps to give important prognostic and predictive information most especially with clinical decision making during multidisciplinary meetings. For example, many studies support the view that adjuvant, single-agent fluoropyrimidine-based chemotherapy is less beneficial, or even potentially harmful, for patients with MSI-H [20]. In a study in Singapore looking at the prevalence of incidental colorectal cancer at autopsy and future trends, the tumours were detected in the older population, and this was similar to the patient in this case report. The majority of the tumours occurred in the ascending colon, similar to our patient who had the tumour in the ascending colon. Cancers in this location are known to produce symptoms late in the course of the disease, due to the larger size of the intestinal lumen, and the increased fluidity of the faecal load. This distribution of incidental tumours in the right colon could also be attributed to the fact that left colon cancers tend to cause symptoms earlier and are more likely to be detected during life than right-sided tumours. This has an important significance as the true incidence of right-sided colon cancers is most likely to be underestimated in the UK [12].

## Conclusions

Colorectal cancer is a common malignancy and not uncommonly asymptomatic. This case presentation highlights the importance of careful and systematic gross and patho-morphological examination of the non-cancer bowel specimen as it may harbor early malignancy, especially in higher-risk groups.
